# Acute multifocal retinitis: a retrospective review of 35 cases

**DOI:** 10.1186/s12348-018-0160-9

**Published:** 2018-10-17

**Authors:** Sana Khochtali, Salma Gargouri, Sourour Zina, Imen Ksiaa, Nesrine Abroug, Sonia Zaouali, Bechir Jelliti, Sonia Attia, Moncef Khairallah

**Affiliations:** 1 Department of Ophthalmology, Faculty of Medicine, Fattouma Bourguiba University Hospital, University of Monastir, Monastir, Tunisia; 2 Department of Ophthalmology, Faculty of Medicine, Habib Bourguiba University Hospital, University of Sfax, Sfax, Tunisia

**Keywords:** Posterior uveitis, Retinitis, Branch retinal artery occlusion, Rickettsial disease, Cat-scratch disease

## Abstract

**Background:**

Acute multifocal retinitis is a rare condition that has been considered to be often idiopathic. The purpose of this study was to analyze clinical features and causes of acute multifocal retinitis.

**Results:**

This study is a retrospective review of the charts of 35 patients with acute multifocal retinitis. Patients with three or more retinal lesions in at least one eye, with at least one lesion of less than 500 μm in size were included. All patients had complete ophthalmological examination, fundus photography, and fluorescein angiography. Twelve patients (34.3%) had optical coherence tomography. An extensive work-up was performed including a detailed comprehensive medical history, examination by an internist and an infectious disease specialist, a chest X-ray, Mantoux test, and laboratory tests for syphilis, human immunodeficiency virus, Bartonella, and Rickettsia. Of the 35 patients, 25 (71.4%) had bilateral involvement and 10 (28.6%) had unilateral involvement (total number of eyes: 60). Mean best-corrected visual acuity (BCVA) was 20/25 (range, 20/1000–20/20). Retinal lesions ranged from 3 to more than 20 in number in at least 1 eye, and from 150 to 1500 μm in size. Associated findings included mild anterior chamber inflammation in 5 eyes (8.3%), mild vitritis in 46 eyes (76.7%), optic disc swelling in 9 eyes (15%), macular star in 4 eyes (6.7%), exudative retinal detachment in 6 eyes (10%), and branch retinal artery occlusion in 6 eyes (10%). Acute multifocal retinitis was found to be caused by *Rickettsia conorii* infection in 20 patients (57.1%), *Rickettsia typhi* infection in 4 patients (11.4%), cat-scratch disease in 8 patients (22.9%), and syphilis in 1 patient (2.9%). It was idiopathic in two patients (5.7%). Retinal lesions resolved without scarring in 3 to 12 weeks in all but three eyes (5%), in which residual retinal pigment epithelial changes were noted.

**Conclusion:**

Rickettsial disease was the most common cause of acute multifocal retinitis. Other identified causes included cat-scratch disease and syphilis, and a very small subset of patients was diagnosed with idiopathic multifocal retinitis.

## Background

Acute multifocal retinitis (AMR), usually preceded by a flu-like illness, is a rare condition usually affecting otherwise healthy adults, characterized by the occurrence of multiple small or mid-sized superficial retinal infiltrates. These lesions have typically a self-limited course with patients regaining a normal visual acuity [1–3]. AMR was first described by Goldstein and Pavan in ten eyes of six patients in 1985 [1]. Since then, a few cases have been reported [2–6]. This condition has been considered to be often idiopathic and sometimes secondary to cat-scratch disease or syphilis [3, 5, 6]. However, as most patients develop a systemic flu-like illness, another yet “unrecognized” blood-borne viral or bacterial infection has been suggested to be a possible causative agent of AMR [3–5].

The purpose of our study was to analyze clinical findings and etiologies in a large cohort of patients with AMR.

## Methods

The medical records of 35 patients diagnosed with AMR at Fattouma Bourguiba University Hospital, Monastir, Tunisia, from January 1, 2003 to January 31, 2017 were retrospectively reviewed. Patients with three or more retinal lesions in at least one eye, with at least one lesion of less than 500 μm in size were included. Patients with a history of diabetes, hypertension, hematological disorders, or any known retinal vascular disease were excluded.

All patients underwent a complete ophthalmological examination including the assessment of best-corrected visual acuity (BCVA), slit-lamp examination, tonometry, and dilated biomicroscopic fundus examination. Fundus photography and fluorescein angiography were also performed in all cases. Twelve patients (34.3%) had optical coherence tomography. An extensive work-up was performed including a detailed comprehensive medical history taking, examination by an internist and an infectious disease specialist, chest X-ray, complete blood cell count, erythrocyte sedimentation rate, C*-*reactive protein, antinuclear antibody, Mantoux test, and laboratory testing for syphilis, human immunodeficiency virus, cat scratch disease, and rickettsial infection.

The mean follow-up period was 8.5 months (range, 6–12). Statistical analysis was performed with the paired Student *t* test exact test. A *p* value less than 0.05 indicated statistical significance.

## Results

Mean age of our patients was 38 years (range: 17–59). Of 35 patients, 14 (40%) were female, and 21 (60%) were male. Thirty patients (85.7%) reported a flu-like illness 4 to 21 days before the onset of ocular symptoms. The most frequent systemic manifestations included fever in 30 patients (85.7%), malaise in 23 patients (65.7%), upper respiratory symptoms in 12 patients (34.3%), and skin rash in 8 patients (22.9%). One patient (2.9%) was diagnosed with lymphocytic meningitis by internist.

Sixty eyes of 35 patients were included (25 patients with bilateral ocular involvement (71.4%) and 10 (28.6%) with unilateral involvement). Ocular symptoms included floaters in 20 patients (57.2%), vision blurring in 13 patients (37.1%), and scotomata in 2 patients (5.7%).

The BCVA at presentation ranged from 20/20 (66.7%) to 20/1000 (2.6%), with a mean BCVA of 20/25.

The anterior chamber was quiet in 55 eyes (91.7%). Five eyes (8.3%) showed 1 to 2+ cells in the anterior chamber. A mild vitritis was found in 46 eyes (76.7%).

**Fig. 1:**
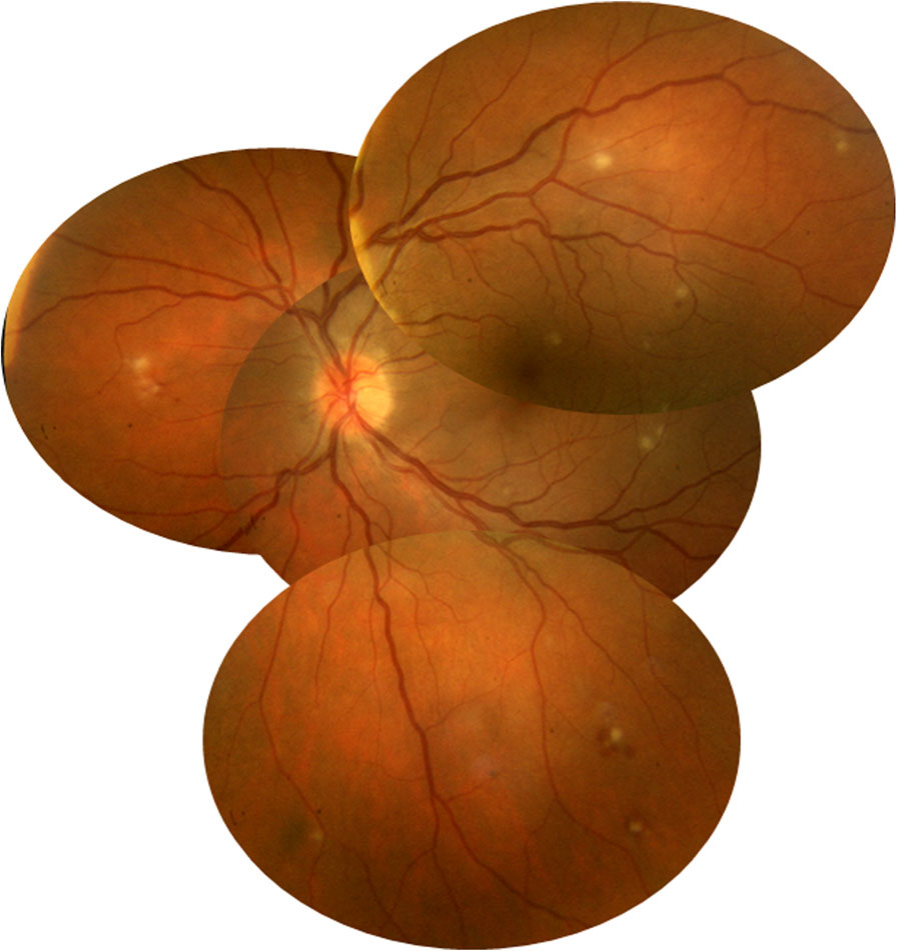
A 42-year-old patient complained of floaters. He reported a 2-week history of fever and malaise. Composite fundus photograph of the left eye shows multiple small white retinal lesions in the posterior pole and the periphery, with a few retinal hemorrhages. Laboratory testing was positive for Mediterranean spotted fever (*Rickettsia Conorii* infection)

**Fig. 2:**
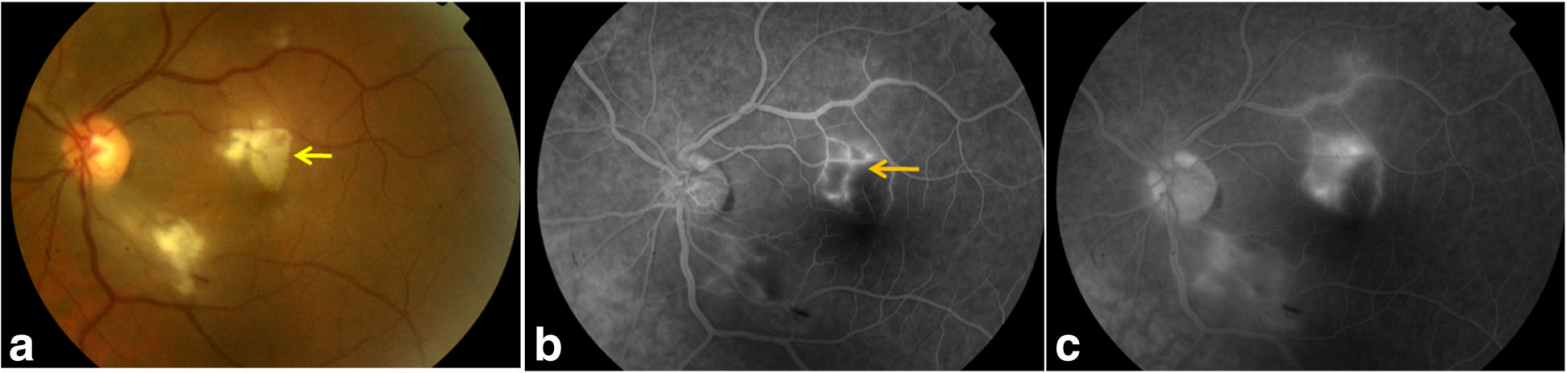
A 38-year-old male patient presented with acute scotoma in the left eye. Color fundus photograph shows three retinal infiltrates with a triangle-shaped area of retinal ischemic whitening adjacent to a retinal infiltrate (arrow) (**a**). Fluorescein angiography confirms the presence of branch retinal artery occlusion corresponding to the area of retinal ischemic whitening (arrow). It also shows early hypofluorescence of the larger infiltrates and isofluorescence of the smaller infiltrate (**b**) and late staining of all three retinal infiltrates, retinal vascular leakage, and optic disc hyperfluorescence (**c**). Laboratory testing was positive for Mediterranean spotted fever (*Rickettsia Conorii* infection)

**Fig. 3:**
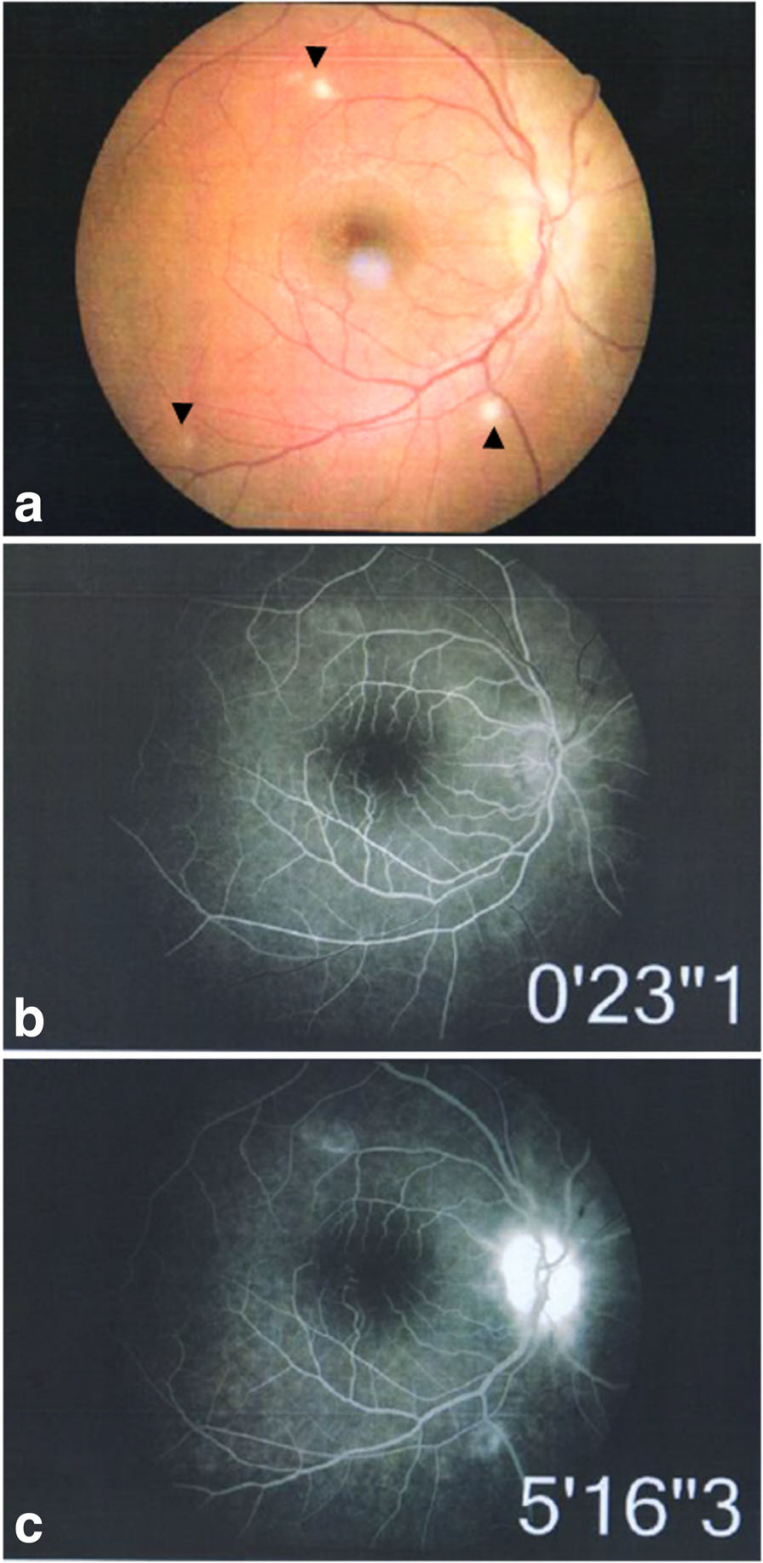
A 32-year-old female patient presented with vision blurring in the right eye. The patient reported having been recently scratched by a kitten. There was a right relative afferent pupillary defect. Color fundus photograph shows retinal infiltrates (arrowheads) (**a**). Fluorescein angiography shows mild peripheral staining of retinal lesions (**b**, **c**) and late optic disc hyperfluorescence (**c**). Serology confirmed a diagnosis of cat-scratch disease

**Fig. 4:**
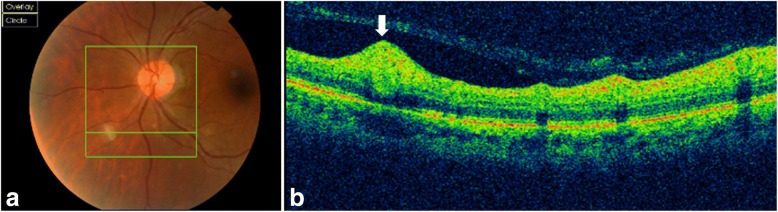
A 38-year-old female patient complained of floaters OU. Color fundus photograph shows three white retinal infiltrates, inferior to the optic disc (**a**). Spectral-domain OCT image section passing through the largest lesion reveals a focal area of retinal thickening with hyperreflectivity extending from the retinal nerve fiber layer to the outer nuclear layer (white arrow), sparing of the retinal pigment epithelium and a mild posterior shadowing (**b**). Laboratory testing was positive for murine typhus (*Rickettsia typhi* infection)

**Fig. 5:**
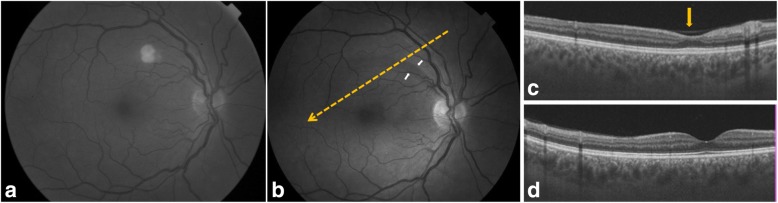
A 43-year-old patient complained of floaters, OU. Red-free fundus photograph of the right eye shows a mid-sized white retinal infiltrate in the posterior pole (**a**). Serology confirmed Mediterranean spotted fever. Red-free fundus photograph, taken 3 months after presentation, shows resolution of the retinal infiltrate with development of a retinal nerve fiber layer defect demarcated by the white arrows (**b**). Spectral-domain OCT image section passing through the clinically visible localized retinal nerve fiber layer defect (yellow dashed arrow in **b**) shows an indentation (arrow) corresponding to a thinning of the retinal nerve fiber layer (**c**). Spectral-domain OCT image section passing through the foveola shows normal findings (**d**)

**Fig. 6:**
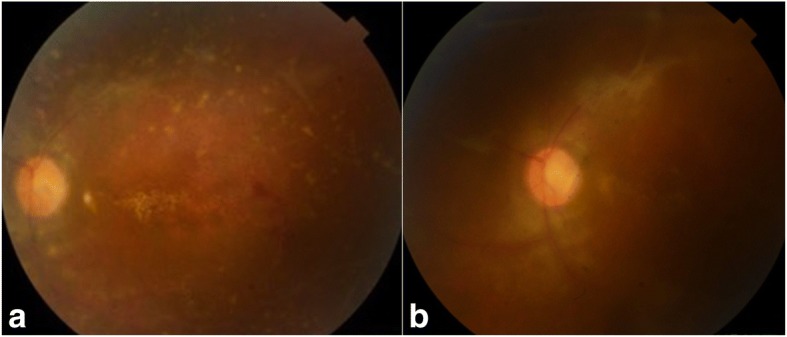
A 23-year-old female patient presented with vision blurring in the left eye. Color fundus photograph shows numerous white retinal lesions, associated with diffuse venous sheathing and a few retinal hemorrhages (**a**). Serology was positive for syphilis. Color fundus photograph, 2 months after presentation, shows residual retinal pigment epithelial changes and venous sheathing (**b**)

Fundus examination revealed white retinal infiltrates in all 60 eyes (100%). Lesions were located in the posterior pole in 15 eyes (25%), in the retinal periphery in 15 eyes (25%), and in both the posterior pole and the retinal periphery in 30 eyes (50%). The number of the white lesions varied from 1 (3 eyes [5%]) to more than 20 (1 eye [1.7%]). Lesion size ranged from 150 to 1500 μm. Small lesions appeared to be superficial, and large lesions appeared denser involving the retina more deeply (Figs. 1, 2, 3, 4, 5, and 6). On fluorescein angiography, the white retinal lesions were either slightly hypofluorescent or isofluorescent without staining, or stained at the late phase (Figs. 2 and 3). On OCT, these lesions showed focal hyperreflectivity and thickening primarily involving the inner retinal layers with or without associated posterior shadowing (Fig. 4).

Associated findings included optic disc swelling in 9 eyes (15%), optic disc hyperfluorescence in 42 eyes (70%), retinal hemorrhages in 23 eyes (38.3%), macular star in 4 eyes (6.7%), macular exudative retinal detachment in 6 eyes (10%), branch retinal artery occlusion (BRAO) in 6 eyes (10%), and retinal vascular leakage in 8 eyes (13.3%).

Causes of AMR included Mediterranean spotted fever (serologically proven *Rickettsia conorii* infection) in 20 patients (57.1%), murine typhus (serologically proven *Rickettsia typhi* infection) in 4 patients (11.4%), cat-scratch disease in 8 patients (22.9%), and syphilis in 1 patient (2.9%). Work-up results were negative in two patients (5.7%).

Thirty-four patients (97.1%) were given an oral antibiotic therapy including a 2- to 4-week course of oral doxycycline in 32 patients (91.4%) and a 2-week course of ciprofloxacin in 2 patients (5.7%). The patient with ocular syphilis received intravenous cefotaxime (2 g six times a day) for 14 days.

Systemic manifestations had a favorable outcome in all cases. White retinal lesions disappeared in 3 to 12 weeks, in 57 eyes (95%). Three eyes (5%) had residual retinal pigment epithelial changes (Figs. 5 and 6). Color fundus photography during follow-up (only available in a subset of patients) revealed a localized retinal nerve fiber layer defect resulting from resolved retinal infiltrates in the posterior fundus in five eyes. OCT sections passing through the clinically visible localized retinal nerve fiber layer defect revealed an indentation corresponding to a thinning of the retinal nerve fiber layer (Fig. 5).

Final BCVA ranged from 20/20 (86.7%) to 20/800(3.3%). The improvement of BCVA was statistically significant (*p* = 0.008). Two patients had a final BCVA of less than 20/200 due to subfoveal inflammatory choroidal neovascular membrane in one patient with rickettsial disease and to a macular atrophy in the patient with ocular syphilis. No recurrence of multifocal retinitis was recorded in any patient.

## Discussion

This case series of 35 patients (60 eyes) with AMR is the largest reported to date. The clinical characteristics of AMR in our patients were similar to that described in previous studies. Flu-like illness occurred in 85.7% of the patients in our study, 1 to 3 weeks prior to the onset of visual symptoms. Such manifestations have been previously reported in one half to all patients with AMR [1–6]. Results of our study, consistent with previous data, show that retinal infiltrates tended to be bilateral, scattered in the posterior pole and/or the retinal periphery. Lesion size ranged from 150 to 1500 μm. Small lesions appeared to involve the inner retinal layers, and large lesions seemed to involve all retinal layers [1–6]. The retinal pigment epithelium tended to be spared on OCT in most affected eyes [5]. Small white retinal lesions might simulate cotton-wool ischemic spots. However, their distribution in the fundus was not necessarily associated with the distribution of a first-order arteriole, and FA did not show retinal capillary non-perfusion in their location, as is the case with cotton-wool spots [3, 5, 7].

On the other hand, an array of other posterior segment changes accompanied AMR in our patients including mild vitritis in 76.7% of eyes, optic disc swelling in 15% of eyes, exudative retinal detachment and branch retinal artery occlusion in 10% of eyes each, and macular star in 6.7% of eyes. These findings are consistent with previous descriptions of AMR [1–6].

In contrast to previous studies showing a high rate of idiopathic AMR, work-up was negative in only two of our patients (5.7%) [1–6]. AMR was associated with cat-scratch disease in a subset of our patients, which is consistent with literature [6, 8]. Our results indicate that rickettsial disease was by far the most common etiology of AMR. To the best of our knowledge, the entity AMR with or without a prodromal flu-like illness has not been previously associated with rickettsial infection. In literature, rickettsial serology was included in the work-up of only one patient with AMR and was negative in that case [4].

On the other hand, superficial white retinal lesions similar to that seen in AMR were reported to be a common and typical clinical finding in rickettsial disease including Mediterranean spotted fever, murine typhus, and rocky mountain spotted fever [9–13].

Focal retinal infiltrates associated with rickettsial infection, cat-scratch disease, or syphilis may result directly from intraretinal bacterial multiplication, or from immune-mediated response to bacterial antigens caused by the deposition of immune complexes, inflammatory cells, or antibodies [3, 9].

Multifocal white retinal lesions occurring after a febrile illness may also be related to Chikungunya or dengue fever in patients living in or returning from specific endemic areas [14]. Differential diagnoses also include Behçet uveitis, viral disease, toxoplasmosis, and “white dot syndromes” [3–5]. All these diagnoses were excluded in our patients by a comprehensive history and ophthalmological evaluation, systemic examination, and tailored laboratory investigations.

Most of our patients recovered a normal visual acuity. The limited recovery seen in two eyes of two patients was attributed to macular atrophy and subfoveal choroidal neovascularization respectively. White retinal lesions disappeared completely within 3 to 12 weeks, in all but three eyes that developed residual retinal pigment epithelial changes. Complete resolution without visible chorioretinal scarring and good visual outcome is typical of AMR in previous data [1–6]. A subset of our patients developed localized retinal nerve fiber layer defects as sequelae of retinitis foci. Such defects without visible scarring have previously been described after the resolution of active infiltrates in Behçet uveitis and in Chikungunya virus infection [15, 16].

## Conclusions

In summary, our results show that rickettsial disease should be highly suspected in patients with AMR, with or without a prior systemic flu-like illness. Rickettsioses are worldwide distributed zoonoses with variable geographical distribution. Therefore, an appropriate and comprehensive serological testing tailored to local epidemiological data is recommended not to miss an underlying rickettsial disease in patients with AMR.

## Abbreviations

AMR: Acute multifocal retinitis
BCVA: Best-corrected visual acuity
OCT: Optical coherence tomography
